# A meta-analysis of the placebo response in antimuscarinic drug trials for overactive bladder

**DOI:** 10.1186/1471-2288-9-55

**Published:** 2009-07-22

**Authors:** Soyon Lee, Bimal Malhotra, Dana Creanga, Martin Carlsson, Paul Glue

**Affiliations:** 1 Ernest Mario School of Pharmacy, Rutgers University, New Brunswick, NJ, USA; 2 Clinical Pharmacology Department, Pfizer Inc, New York, NY, USA; 3 Statistics Department, Pfizer Inc, New York, NY, USA; 4 Dunedin School of Medicine, Dunedin, New Zealand

## Abstract

**Background:**

The purpose of this analysis was to characterize the placebo response in antimuscarinic drug trials for OAB, based on changes in commonly-used efficacy endpoints.

**Methods:**

Placebo arm data for incontinence episodes, micturitions, voided volume and study characteristics were extracted from randomized placebo controlled antimuscarinic drug trials in OAB, from studies identified in a prior meta-analysis, and from a systematic review of more recently published studies. Relationships between variables were examined using linear regression, and changes in endpoints were analyzed by a meta-analysis approach. The effect of placebo arm size and magnitude of placebo response on probability of successful study outcome was analyzed using an ANOVA model.

**Results:**

Changes in the placebo arms for all 3 endpoints were substantial and statistically significant, and highly heterogeneous. There were significant associations between baseline and change scores for some but not all of the endpoints. More recent studies tended to have more subjects than earlier studies, and there were positive associations between probability of achieving statistically significant results and size of the placebo arm. The magnitude of changes in placebo arms did not appear to influence the likelihood of the study to be statistically significant.

**Conclusion:**

This analysis confirms earlier observation that the placebo response in OAB trials is substantial and highly heterogeneous. There are multiple potential reasons for this; however, these could not be explored in this analysis of study-level data. Two approaches may be used in clinical trials to manage high placebo effect: recruitment of 1) greater numbers of patients and/or 2) more severely affected patients; however, only the former approach is associated with increased probability of successful study outcome.

## Background

Clinical trials of antimuscarinic drugs in overactive bladder (OAB) have noted marked responses in patients treated with placebo. While this led some to question the usefulness of treatment interventions [1,2], more recent reviews have confirmed the clinical benefit of antimuscarinics in OAB [3,4]. However both of these metaanalyses also emphasized the modest difference in outcome measures between active and placebo arms. Nabi et al [3] calculated that 41% of subjects allocated to placebo arms reported symptomatic improvement or cure, compared with approximately 56% in patients allocated to active treatment. Chapple et al [4] also noted considerable variation in placebo rates between trials.

Despite these observations, the placebo response in drug trials for OAB has not been well characterized. One paper reported on study level data in registration trials for drugs treating lower urinary tract symptoms [5] and another from patient level data in four pooled studies in stress urinary incontinence [6]. Issues identified with high placebo response included patient and disease characteristics (e.g. less severe disease), amount of prior and concomitant treatment (e.g. pelvic floor training), and types of endpoints (subjective rather than objective). Nonspecific factors associated with trial participation, such as increased awareness of voiding habits and interactions with clinical trial staff were also considered relevant.

The purpose of this analysis was to characterize the placebo response in antimuscarinic drug trials for OAB, based on changes in commonly-used efficacy endpoints of 1) number of micturitions per day, 2) number of incontinence episodes per day and 3) mean voided volume per micturition. A number of statistical methods were used, including a meta-analysis to obtain a more precise estimate of the placebo effect based on pooled results from various studies.

## Methods

### Data Sources and Study Selection

**Figure 1:**
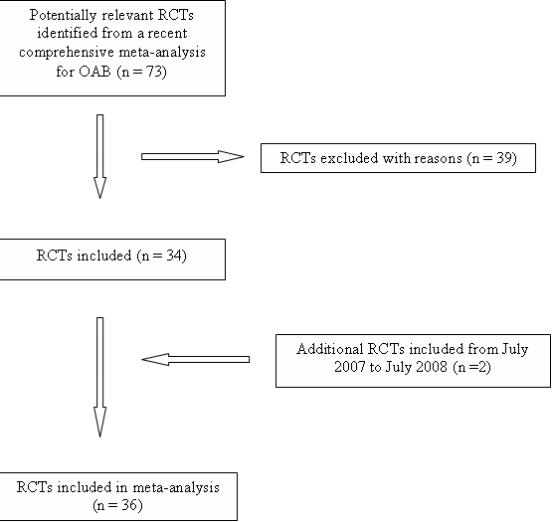
Search Strategy for Selection of Randomized Clinical Trials for Meta-Analysis.

The search strategy for the selection of randomized clinical trials for the meta-analysis of placebo response is summarized in Figure 1. We selected all placebo-controlled trials included in a recent comprehensive meta-analysis of antimuscarinic treatments for OAB [7]. In addition, we identified any additional trials that had been published in the 12 months up to July 2008 through a systematic review of the literature using all major literature databases for publications reporting randomized, double-blinded, placebo-controlled trials for individuals diagnosed with OAB, and that were not included in the recent meta-analysis [7]. Data collection, selection, extraction and recording were in accordance with Cochrane Reviews Guidelines [8]. Searches included combinations of the terms **overactive bladder, urge urinary incontinence, randomized, double-blind, placebo-controlled**, and **names of common antimuscarinic medications**. In addition, searches included the abstracts from several major conferences. There were seven drugs included in this review (darifenacin, fesoterodine, oxybutynin, propiverine, solifenacin, tolterodine, and trospium).

For inclusion in this analysis, publications had to meet the following eligibility criteria; 1) the study was a double-blind randomized placebo-controlled trial of an antimuscarinic medication in patients with OAB; 2) the total number of patients assigned to placebo was reported, and 3) the study reported one or more of the following endpoints: number of incontinence episodes per day, number of micturitions per day, and/or volume voided per micturition. Additional information was requested through direct contact with study authors for abstracts that did not report some of the above details, or for studies where endpoint data were presented as medians rather than means. Because of different assumptions about data distribution, only studies reporting mean data were included.

### Study Procedure (Data Extraction)

After the publications were obtained, two of the authors (SL and PG) independently determined the eligibility of each publication by applying the above criteria. If data were reported in more than one publication, only the primary publication was included in this analysis. Study characteristics (year of publication, patient numbers, ages, duration of treatment, diary details), and baseline, endpoint, and change from baseline of the above endpoints, associated estimates of variability (if reported) and statistical power were extracted by one author and entered into Microsoft Excel. Studies that reported their endpoints as number of incontinence episodes per week were converted to episode per day, by dividing the values by seven.

### Statistical Methods

Summary statistics were calculated for all variables. The relationship between baseline and change in endpoints, and study size versus year of publication, was assessed using linear regression. The probability of success (achieving statistical significance) as a function of the size of the placebo arm was estimated using logistic regression. The relationship between year of publication and baseline and change in endpoints was assessed using Spearman regression. A meta-analysis was conducted to pool the results of change from baseline data for each of the 3 endpoints using Comprehensive Meta-analysis software version 2.0. For each endpoint, change from baseline was summarized as non-standardized (weighted) means using inverse variance weighting. Point estimates and 95% confidence intervals (CIs) were computed with both random effects (Der Simmonian and Laird method) and fixed effects models. The null hypothesis of homogeneity of response across studies was tested with the Cochran Q statistic. If the null hypothesis was rejected, point estimates and 95% CI were estimated on the basis of random effects model was presented, otherwise the fixed effects model was presented. An ANOVA model was used to assess the possibility that the magnitude of placebo change might influence the probability of study success, with magnitude of change in the placebo arms as the dependent variable and study, success (statistically significant separation between active and placebo arms) and power of the study (80% or 90%) as independent variables. Statistical testing was carried out at the 5% level of significance (two-sided tests).

## Results

### Trial characteristics

Thirty-four publications which included a placebo arm were identified in the Chapple meta-analysis [7]. The systematic literature review over the most recent 12 months identified 2 additional studies [9,10]. The 36 studies were identified that met acceptance criteria, and these are listed in Table 1[9-44]. The most commonly published OAB trials were for tolterodine (n = 15), oxybutynin (n = 8), propiverine (n = 5) and solifenacin (n = 5) (note: several studies included more than one active arm). The mean age of patients enrolled in the placebo arms was 58.9 years. All studies included adult subjects but four studies specifically targeted elderly subjects [12,19,21,30]. Median study duration was 12 weeks (range 2–12 weeks). The mean number of patients in the placebo arms of the trials was 164 (range 13–508).

**Table 1:** Results for placebo treatments in the studies included in the meta-analysis.

Author, date [reference]	Placebo	Duration (wks)	Mean micturitions/day			Mean incontinence episodes/day			Mean volume voided per micturition (mL)		
	**n**		**BL**	**EOT**	**CFBL**	**BL**	**EOT**	**CFBL**	**BL**	**EOT**	**CFBL**

Abrams, 1998 [11]	57	12	11.7	NR	-1.6	3.3	NR	-0.9	157	NR	6

Burgio, 1998 [12]	65	8	NR	NR	NR	2.2	1.2	-1.03	NR	NR	NR

Cardozo, 2004 [13]	301	12	NR	NR	-1.59	NR	NR	-1.25	NR	NR	10.67

Chapple, 2004 [14]	38	4	11.1	10.1	-1.03	1.7	1.4	-0.29	134.7	144.4	9.7

Chapple, 2004 [15]	267	12	12.2	11	-1.2	2.7	2	-0.76	143.8	151.2	7.4

Chapple, 2007 [16]	285	12	12	10.9	-1.02	3.7	2.5	-1.2	150.2	159.9	9.77

Dmochowski, 2002 [17]	132	12	NR	NR	-1.7	NR	NR	NR	NR	NR	NR

Dmochowski, 2003 [18]	117	12	12.3	10.9	-1.4	5	2.9	-2.1	175	182	9

Dmochowski, 2008 [9]	284	12	12.9	11.1	-1.8	4	2.4	-1.6	151.8	169.6	17.8

Dorschner, 2003 [19]	49	4	7.1	6.5	-0.6	0.4	0.2	-0.1	187	178	-8.4

Drutz, 1999 [20]	56	12	11.4	10.3	-1.1	3.6	2.6	-1	160	172	12

Halaska, 1994 [21]	47	4	NR	NR	NR	NR	NR	NR	195	221	26

Herschorn, 2007 [22]	204	12	11.8	10.1	-1.7	3.2	1.8	-1.4	NR	NR	NR

Homma, 2003 [23]	122	12	11.1	9.6	-1.5	2.7	1.6	-1.09	130.7	145.8	15.2

Jacquetin, 2001 [24]	51	4	11.7	10.5	-1.2	2.4	NR	-0.4	148	155	7

Junemann, 2000 [25]	60	3	NR	NR	-1.9	NR	NR	NR	NR	NR	NR

Junemann, 2006 [26]	202	4.57	13.4	10.3	-3.07	3.5	1.7	-1.78	144.2	173.5	29.3

Khullar, 2004 [27]	285	8	10.6	9.3	-1.3	3.1	NR	-1.14	167	185.9	18.9

Lee 2006, [28]	79	12	13	10.4	-2.58	NR	NR	NR	NR	NR	NR

Madersbacher, 1999 [29]	72	4	11.5	10.5	-1	NR	NR	NR	NR	NR	NR

Malone-Lee, 2001 [30]	43	4	9.9	10.3	0.4	5.1	4.4	-0.7	152	162	10

Malone-Lee, 2002 [31]	73	12	NR	NR	NR	NR	NR	NR	NR	NR	15.91

Millard, 1999 [32]	64	12	11.3	9.9	-1.4	3.5	2.2	-1.3	158	168	10

Nitti, 2007 [33]	274	12	12.2	11.1	-1.08	3.7	2.7	-0.96	159	167.4	8.38

Rackley, 2006 [34]	421	12	12.6	NR	NR	0.72	NR	NR	140.1	NR	NR

Rentzhog, 1998 [35]	13	2	10.2	NR	-0.3	4.1	NR	-0.4	NR	NR	NR

Robinson, 2007 [36]	61	6	11.9	10.1	-1.81	2.9	2.2	-0.66	145.5	156.9	11.4

Rogers, 2008 [37]	211	12	12.5	10.3	-2.2	2.2	0.8	-1.3	NR	NR	NR

Rudy, 2006 [38]	329	12	13.2	11.4	-1.76	NR	NR	NR	154.6	164.1	9.44

Staskin, 2007 [10]	303	12	12.7	10.8	-1.99	4.1	2.2	-1.93	155.9	174.8	18.89

Thuroff, 1991 [39]	27	4	NR	NR	-0.3	NR	NR	NR	NR	NR	NR

Uchida, 2002 [40]	53	4	10.9	9.9	-1	2.3	1	-1.3	196	202	6

van Kerrebroeck, 2001 [41]	508	12	11.3	9.1	-2.2	3.3	2.3	-0.99	136	150	14

Wang, 2006 [42]	21	12	NR	NR	NR	NR	NR	NR	350	340	10

Yamaguchi, 2007 [43]	405	12	11.4	10.3	-0.94	2	1.3	-0.72	152.8	164.5	11.67

Zinner, 2004 [44]	256	12	12.9	11.6	-1.29	4.3	2.4	-1.9	156.6	164.3	7.7

BL: Baseline

EOT: End of treatment

CFBL: Change from baseline

NR: Not reported

**Figure 2:**
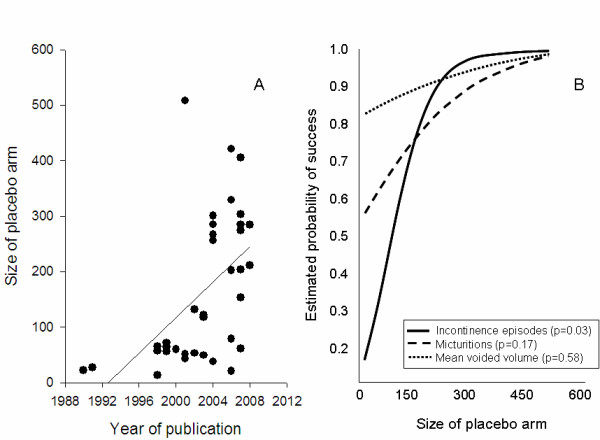
Relationship of the size of placebo arm with (A) the year of publication (Panel A) and the probability of successful study outcome (Panel B) for three commonly used endpoints.

Size of placebo arms tended to increase in more recent studies (r = 0.52; Figure 2A). There were positive associations between the probability of studies reporting statistically successful outcomes and the size of the placebo arm for all endpoints (Figure 2B). This was statistically significant for incontinence episodes (p = 0.03), but not for micturitions/day (p = 0.17) or mean voided volume (p = 0.58). It was not possible to assess the influence of some variables on placebo response, either because they were too homogeneous (e.g. study duration was 12 weeks in 22/36 studies), or because they were not reported (e.g. diary duration in only half of the studies).

### Incontinence episodes/day

**Figure 3:**
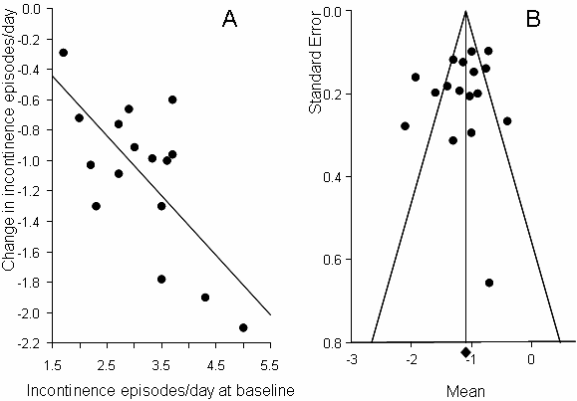
**Incontinence episodes/day.** Panel A: Relationship between baseline and change scores. Panel B: Funnel plot from meta-analysis.

Baseline mean (SD) incontinence episodes/day in subjects randomized to placebo were 3.16 (1.00). At study endpoint, mean (SD) incontinence episodes/day were reduced by 1.16 (0.46). The change in incontinence episodes/day was highly associated with baseline values (r = 0.69; Figure 3A). Although there was no relationship between baseline incontinence episodes and year of publication (r = -0.03), there was a modest positive relationship between change in incontinence episodes and year of publication (r = 0.39, p = 0.10). There was a negative correlation between study size and change in incontinence episodes/day (r = -0.25).

**Figure 4:**
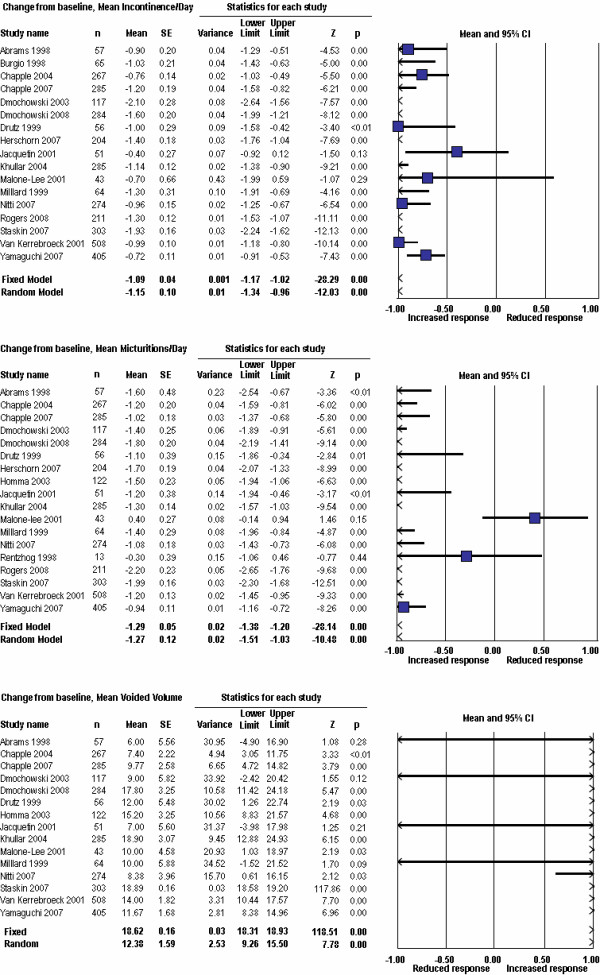
Forest plots form the meta-analysis of commonly used endpoints in OAB trials.

Point estimates (95%CI) from the meta-analysis of change from baseline data were -1.09 (-1.17, -1.02) using a fixed effect model, and -1.15 (-1.34, -0.96) using a random effects model. The forest plot is shown in Figure 4, upper panel. Both results were highly statistically significant (p < 0.0001). Considerable heterogeneity in the data set was suggested by the high Q-value (85.2, df = 16, p < 0.0001), indicating that a random effects model was a more appropriate analytical approach. The high degree of data heterogeneity was also evident on the funnel plot (Figure 3B). Analysis of the relationship between the magnitude of placebo response and successful study outcome and power showed no statistical difference (p = 0.80 and 0.97 respectively).

### Mean micturitions per day

**Figure 5:**
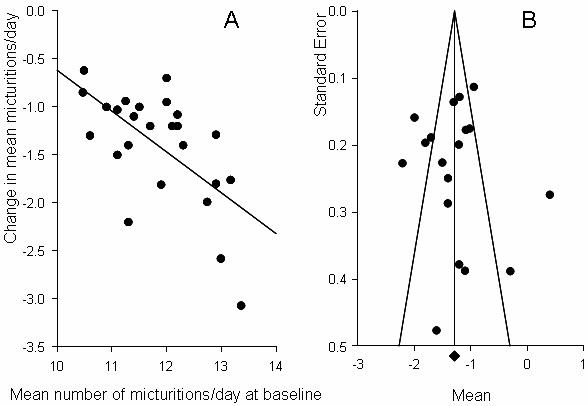
**Mean micturitions/day.** Panel A: Relationship between baseline and change scores. Panel B: Funnel plot from meta-analysis.

Baseline mean (SD) micturitions/day in subjects randomized to placebo were 11.8 (0.9). At study endpoint, mean (SD) micturitions/day were reduced by 1.4 (0.7). The change in mean micturitions/day was highly associated with baseline values (r = 0.62; Figure 5A). There was a modest positive relationship between baseline micturitions/day and year of publication (r = 0.34, p = 0.09), however there was no relationship between change in micturitions/day and year of publication (r = 0.02). There was a modest negative correlation between study size and change in micturitions/day (r = -0.21).

Point estimates (95% CI) from the meta-analysis of change from baseline data were -1.29 (-1.38, -1.12) using a fixed effect model, and -1.27 (-1.51, -1.03) using a random effects model. The forest plot is shown in Figure 4, center panel. Both results were highly statistically significant (p < 0.0001). Considerable heterogeneity in the data set was suggested by the high Q-value (107.0, df = 17, p < 0.0001), indicating that a random effects model was a more appropriate analytical approach. The high degree of data heterogeneity was also evident on the funnel plot (Figure 5B). Analysis of the relationship between the magnitude of placebo response and successful study outcome and power showed no statistical difference (p = 0.93 and 0.21, respectively).

### Mean voided volume

**Figure 6:**
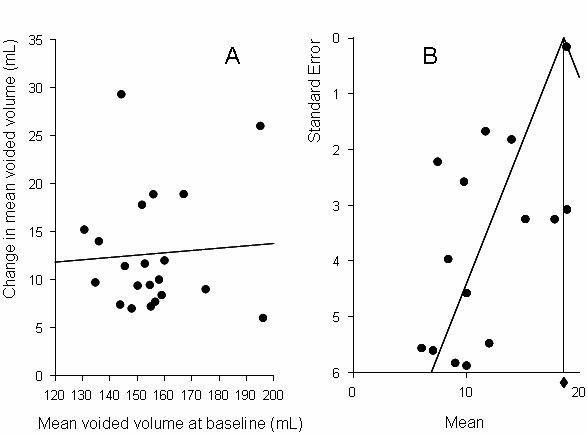
**Mean voided volume/day.** Panel A: Relationship between baseline and change scores. Panel B: Funnel plot from meta-analysis.

Baseline mean (SD) mean voided volume was 163.1 (42.9) mL. After placebo treatment, mean voided volume was increased by 12.5 (5.9) mL. There was no relationship between baseline and change in mean voided volume (r = 0.06; Figure 6A). There was a modest negative relationship between baseline mean voided volume and year of publication (r = -0.23, p = 0.32), however there was no relationship between change in mean voided volume and year of publication (r = 0.06). There was no relationship between study size and change in mean voided volume (r = -0.07).

Point estimates (95% CI) from the meta-analysis of change from baseline data were 18.6 (18.3, 18.9) using a fixed effect model, and 12.4 (9.3, 15.5) using a random effects model. The forest plot is shown in Figure 4, lower panel. Both results were highly statistically significant (p < 0.0001). Considerable heterogeneity in the data set was suggested by the high Q-value (91.0, df = 14, p < 0.0001), indicating that a random effects model was a more appropriate analytical approach. The high degree of data heterogeneity was also evident on the funnel plot (Figure 6B). Analysis of the relationship between the magnitude of placebo response and successful study outcome and power showed no statistical difference (p = 0.26 and 0.50 respectively).

### Interrelationship of endpoints

Changes in all 3 endpoints showed were correlated with one another and showed moderate levels of association. Change in incontinence episodes was positively associated with change in micturitions (r = 0.49). As would be expected based on unchanged daily urine output, the change in mean voided volume was negatively associated with change in incontinence episodes (r = -0.38) and change in micturitions (r = -0.61).

## Discussion

The main findings of this analysis are that for three commonly published endpoints in OAB studies, changes in the placebo arms were substantial and statistically significant. A high degree of heterogeneity was noted for all endpoints. There were significant associations between baseline and change scores for some but not all of the endpoints. More recent studies tended to be larger than earlier studies, and there were positive associations between probability of achieving statistically significant results and size of the placebo arm. The magnitude of changes in placebo arms did not appear to influence the likelihood of the study to be statistically different from active treatment.

This analysis confirms earlier observations that the placebo response in OAB trials is substantial [3,4]. In a meta-analysis of placebo responses across different disorders, drug trials in urogenital disorders had the highest placebo response [45]. This may be an underlying consequence of urological disorders in general, rather than related to type of intervention, as high placebo response rates have also been reported in trials of non-pharmacological management of incontinence. For example, in a trial of pelvic floor muscle training for stress urinary incontinence, a 64% response rate was reported for the sham (placebo) intervention arm [46]. Urological disorders are generally kept private by the patients and a majority of people with UI do not seek help despite poor quality of life (i.e., perceived lack of self-control, limited daily activities for fear of an "accident"). [47] As a result, in routine clinical practice, there may not be a lot of patient-level basic knowledge about these urological conditions. However, when participating in a clinical research trial for a urologic problem, there is a much greater enhancement in patient knowledge and awareness of their disorder than it would be for a condition (e.g., cardiovascular or metabolic) that is more openly discussed.

A meta-analysis approach was used in order to obtain a more precise estimate of the placebo effect based on pooled results from various studies. Both fixed and random effects models were tested, to provide an indication on the variability of the results. Because the meta-analysis revealed a high degree of heterogeneity for all three endpoints, a random effects approach was used in this analysis. Egger et al [48] has identified a number of potential causes of heterogeneity. Factors that might contribute to heterogeneity in OAB studies include the nature of the population studied (i.e. the presence of mixed types of incontinence), size of the studies (ranging from 13 to 508 subjects/arm), use of both subjective and objective endpoints, and/or changes in study methodology and types of patients recruited into OAB trials performed over almost 2 decades. We confirm earlier observations that subjective endpoints may be an important contributor to heterogeneity [5,6]. In OAB trials, a positive correlation of the placebo response with baseline severity was seen for the changes in the endpoints of micturitions and incontinence episodes but was not seen for the mean voided volume.

We were not able to explore the role of other potentially important variable on placebo responses. Because this analysis was performed on study level data, it is not possible to assess the effects of patient level characteristics (e.g. age, gender), or other aspects of design (visit frequency or study location) on responses. It is unclear how important study duration is to placebo responses. Because almost all antimuscarinic OAB studies are 12 weeks long, it is not possible to assess the effects of longer or shorter duration on placebo responses. Finally, the type of diary (paper or electronic) and the duration of recall was not reported by a majority of studies. In particular, the length of time over which patients have to recall subjective endpoint data may be important, with longer durations being associated with greater potential for error.

This analysis confirms that the placebo response in antimuscarinic drug trials for OAB is substantial, and demonstrates high levels of heterogeneity. The substantial placebo response has been noted in treatment trials for other urological disorders, with both drug and non-drug interventions, and may reflect nonspecific effects related to use of a diary, behavioral training, etc, and/or to the use of subjective endpoints. Therefore, in essence, the placebo effect seen in these trials is ascribable to all non-drug aspects of the trial, in addition to treatment with placebo.

Two approaches have been attempted to manage the placebo response- (1) to enroll more severely affected patients in more recent trials (as indicated by the positive association between year of publication and baseline symptom severity); and (2) to enroll larger numbers of subjects. Enrolling more severely affected patients appears to be counterproductive however, as any increase in changes in active treated arms may be offset with larger responses in the placebo arms. Furthermore, our analysis has shown that the probability of study success was unrelated to the magnitude of placebo response in any of the endpoints studied. Using increased sample sizes may have been more effective in ensuring successful study outcome, as demonstrated by the positive associations between these 2 variables (Figure 2B). However this association appears to be greatest for the subjective endpoints, and more modest for the objective endpoint.

## Conclusion

This analysis confirms earlier observations of substantial heterogeneity in placebo responses in antimuscarinic drug trials for OAB. More recent clinical trials have tried to address this by recruiting greater numbers of subjects and/or more severely affected patients; however, only the former approach is associated with increased probability of successful study outcome. Alternative approaches to managing the large and heterogeneous placebo response in OAB drug trials in the future might be to develop and validate more objective endpoints for OAB trials, characterize more drug-responsive subpopulations of patients, and/or to explore different trial designs that can reduce population heterogeneity (e.g. relapse prevention).

## Competing interests

The authors declare that they have no competing interests.

## Authors' contributions

SL collected, analyzed, interpreted the data, and wrote initial drafts of the manuscript. BM contributed to study concept and made substantial contributions to the analysis and interpretation of data. DC conducted a meta-analysis for the 3 endpoints using Comprehensive Meta-analysis software version 2.0. MC performed statistical analyses (Spearman regression, and ANOVA model). PG designed the study, analyzed, and interpreted data and was involved in the drafting and revising of the manuscript. All authors read and approved the final manuscript.

## Pre-publication history

The pre-publication history for this paper can be accessed here:

http://www.biomedcentral.com/1471-2288/9/55/prepub

## References

[B1] Aitchison M Carter R Paterson P Ferrie B Is the treatment of urgency incontinence a placebo response? Results of a five-year follow-up Br J Urol 1989 64 478 80 10.1111/j.1464-410X.1989.tb05280.x 2611617

[B2] Herbison P Hay-Smith J Ellis G Moore K Effectiveness of anticholinergic drugs compared with placebo in the treatment of overactive bladder: systematic review BMJ 2003 326 841 4 12702614 10.1136/bmj.326.7394.841 PMC153465

[B3] Nabi G Cody JD Ellis G Herbison P Hay-Smith J Anticholinergic drugs versus placebo for overactive bladder syndrome in adults Cochrane Database Syst Rev 2006 4 CD003781 17054185 10.1002/14651858.CD003781.pub2 PMC8729219

[B4] Chapple C Khullar V Gabriel Z Dooley JA The effects of antimuscarinic treatments in overactive bladder: a systematic review and meta-analysis Eur Urol 2005 48 5 26 10.1016/j.eururo.2005.02.024 15885877

[B5] van Leeuwen JH Castro R Busse M Bemelmans BL The placebo effect in the pharmacologic treatment of patients with lower urinary tract symptoms Eur Urol 2006 50 440 52 10.1016/j.eururo.2006.05.014 16753253

[B6] Yalcin I Bump RC The effect of previous treatment experience and incontinence severity on the placebo response of stress urinary incontinence Am J Obstet Gynecol 2004 191 194 7 10.1016/j.ajog.2004.03.089 15295364

[B7] Chapple CR Khullar V Gabriel Z Muston D Bitoun CE Weinstein D The effects of antimuscarinic treatments in overactive bladder: an update of a systematic review and meta-analysis Eur Urol 2008 54 543 62 10.1016/j.eururo.2008.06.047 18599186

[B8] Higgins JPT Green S editors Cochrane Handbookfor Systematic Reviews of Interventions Version 5.0.0 [updatedFebruary 2008] 2008 The Cochrane Collaboration http://www.cochrane-handbook.org

[B9] Dmochowski R Sand P Zinner NR Staskin DR Trospium chloride 60 mg once daily (QD) for overactive bladder syndrome: results of a placebo-controlled interventional study Urology 2008 71 449 454 10.1016/j.urology.2007.11.008 18342185

[B10] Staskin D Sand P Zinner N Dmochowski R Once daily trospium chloride is effective and well tolerated for the treatment of overactive bladder: results from a multicenter phase III trial J Urol 2007 178 978 83 10.1016/j.juro.2007.05.058 17632131

[B11] Abrams P Freeman R Anderström C Mattiasson A Tolterodine, a new antimuscarinic agent: as effective but better tolerated than oxybutynin in patients with an overactive bladder Br J Urol 1998 81 801 810 9666761 10.1046/j.1464-410x.1998.00717.x

[B12] Burgio KL Locher JL Goode PS Hardin JM McDowell BJ Dombrowski M Candib D Behavioral vs drug treatment for urge urinary incontinence in older women: a randomized controlled trial JAMA 1998 280 1995 2000 10.1001/jama.280.23.1995 9863850

[B13] Cardozo L Lisec M Millard R van Vierssen Trip O Kuzmin I Drogendijk TE Huang M Ridder AM Randomized, double-blind placebo controlled trial of the once daily antimuscarinic agent solifenacin succinate in patients with overactive bladder J Urol 2004 172 1919 24 10.1097/01.ju.0000140729.07840.16 15540755

[B14] Chapple CR Araño P Bosch JL De Ridder D Kramer AE Ridder AM Solifenacin appears effective and well tolerated in patients with symptomatic idiopathic detrusor overactivity in a placebo-and tolterodine-controlled phase 2 dose-finding study BJU Int 2004 93 71 7 10.1111/j.1464-410X.2004.04561.x 14678372

[B15] Chapple CR Rechberger T Al-Shukri S Meffan P Everaert K Huang M Ridder A YM-905 Study Group: Randomized, double-blind placebo-and tolterodine-controlled trial of the once-daily antimuscarinic agent solifenacin in patients with symptomatic overactive bladder BJU Int 2004 93 303 310 10.1111/j.1464-410X.2004.04606.x 14764127

[B16] Chapple C Van Kerrebroeck P Tubaro A Haag-Molkenteller C Forst HT Massow U Wang J Brodsky M Clinical efficacy, safety, and tolerability of once-daily fesoterodine in subjects with overactive bladder Eur Urol 2007 52 1204 12 10.1016/j.eururo.2007.07.009 17651893

[B17] Dmochowski RR Davila GW Zinner NR Gittelman MC Saltzstein DR Lyttle S Sanders SW For The Transdermal Oxybutynin Study Group: Efficacy and safety of transdermal oxybutynin in patients with urge and mixed urinary incontinence J Urol 2002 168 580 586 10.1016/S0022-5347(05)64684-8 12131314

[B18] Dmochowski RR Sand PK Zinner NR Gittelman MC Davila GW Sanders SW Transdermal Oxybutynin Study Group: Comparative efficacy and safety of transdermal oxybutynin and oral tolterodine versus placebo in previously treated patients with urge and mixed urinary incontinence Urology 2003 62 237 42 10.1016/S0090-4295(03)00356-X 12893326

[B19] Dorschner W Stolzenburg JU Griebenow R Halaska M Brünjes R Frank M Wieners F The elderly patient with urge incontinence or urge-stress incontinence–efficacy and cardiac safety of propiverine Aktuelle Urol 2003 34 102 8 10.1055/s-2003-38906 14566693

[B20] Drutz HP Appell RA Gleason D Klimberg I Radomski S Clinical efficacy and safety of tolterodine compared to oxybutynin and placebo in patients with overactive bladder Int Urogynecol J Pelvic Floor Dysfunct 1999 10 5 283 9 10.1007/s001929970003 10543335

[B21] Halaska M Dorschner W Frank M Treatment of urgency and incontinence in elderly patients with propiverine hydrochloride Neurourol Urodyn 1994 13 428 30

[B22] Herschorn S Heessakkers J Castro-Diaz D Tolterodine extended release (TER) improves objective and subjective outcomes after 1 week of treatment in patients with overactive bladder (OAB) [abstract] Int Urogynecol J 1994 18 Suppl 1 S78

[B23] Homma Y Paick JS Lee JG Kawabe K Clinical efficacy and tolerability of extended-release tolterodine and immediate-release oxybutynin in Japanese and Korean patients with an overactive bladder: a randomized, placebo-controlled trial BJU Int 2003 92 741 7 10.1046/j.1464-410X.2003.04468.x 14616458

[B24] Jacquetin B Wyndaele J Tolterodine reduces the number of urge incontinence episodes in patients with an overactive bladder Eur J Obstet Gynecol Reprod Biol 2001 98 97 102 10.1016/S0301-2115(00)00561-3 11516807

[B25] Junemann KP Al-Shukri S Efficacy and tolerability of trospium chloride and tolterodine in 234 patients with urge-syndrome: a double-blind, placebo-controlled, multicentre clinical trial Neurourol Urodyn 2000 19 488 90

[B26] Jünemann KP Hessdörfer E Unamba-Oparah I Berse M Brünjes R Madersbacher H Gramatté T Propiverine hydrochloride immediate and extended release: comparison of efficacy and tolerability in patients with overactive bladder Urol Int 2006 77 334 9 10.1159/000096338 17135784

[B27] Khullar V Hill S Laval KU Schiøtz HA Jonas U Versi E Treatment of urge-predominant mixed urinary incontinence with tolterodine extended release a randomized, placebo-controlled trial Urology 2004 64 269 75 10.1016/j.urology.2004.02.029 15302476

[B28] Lee KS Choo MS Paick JS Lee JG Seo JT Lee JZ Yoon H Park CH Na Y Jeong YB Lee JB Park WH Propiverine hydrochloride reduced frequency and perception of urgency in treatment of overactive bladder: a 12 week prospective, randomized, double-blind, placebo controlled study Paper presented at: Annual Meeting of the International Continence Society; November 29 – December 1, 2006; Christchurch, New Zealand

[B29] Madersbacher H Halaska M Voigt R Alloussi S Hofner K A placebo-controlled, multicentre study comparing the tolerability and efficacy of propiverine and oxybutynin in patients with urgency and urge incontinence BJU Int 1999 84 646 51 10.1046/j.1464-410x.1999.00251.x 10510109

[B30] Malone-Lee JG Walsh JB Maugourd MF Tolterodine: a safe and effective treatment for older patients with overactive bladder J Am Geriatr Soc 2001 49 700 5 10.1046/j.1532-5415.2001.49144.x 11454106

[B31] Malone-Lee J Whately-Smith C A study of the significance of identifying detrusor instability in the treatment of overactive bladder symptoms Proceedings of the International Continence Society (ICS), 32nd Annual Meeting, Heidelberg, Germany 2002 228

[B32] Millard R Tuttle J Moore K Susset J Clarke B Dwyer P Davis BE Clinical efficacy and safety of tolterodine compared to placebo in detrusor overactivity J Urol 1999 161 1551 5 10.1016/S0022-5347(05)68951-3 10210394

[B33] Nitti VW Dmochowski R Sand PK Forst HT Haag-Molkenteller C Massow U Wang J Brodsky M Bavendam T Efficacy, safety, and tolerability of fesoterodine in subjects with overactive bladder J Urol 2007 178 2488 94 10.1016/j.juro.2007.08.033 17937959

[B34] Rackley R Weiss JP Rovner ES Wang JT Guan Z 037 STUDY GROUP Nighttime dosing with tolterodine reduces overactive bladder-related nocturnal micturitions in patients with overactive bladder and nocturia Urology 2006 67 731 6 10.1016/j.urology.2005.10.061 16618562

[B35] Rentzhog L Stanton SL Cardozo L Nelson E Fall M Abrams P Efficacy and safety of tolterodine in patients with detrusor instability: a dose-ranging study Br J Urol 1998 81 42 8 9467475 10.1046/j.1464-410x.1998.00501.x

[B36] Robinson D Cardozo L Terpstra G Bolodeoku J A randomized double-blind placebo-controlled multicentre study to explore the efficacy and safety of tamsulosin and tolterodine in women with overactive bladder syndrome BJU Int 2007 100 840 5 10.1111/j.1464-410X.2007.07162.x 17822465

[B37] Rogers R Bachmann G Jumadilova Z Sun F Morrow JD Guan Z Bavendam T Efficacy of tolterodine on overactive bladder symptoms and sexual and emotional quality of life in sexually active women Int Urogynecol J Pelvic Floor Dysfunct 2008 19 1551 7 10.1007/s00192-008-0688-6 18685795

[B38] Rudy D Cline K Harris R Goldberg K Dmochowski R Multicenter phase III trial studying trospium chloride in patients with overactive bladder Urology 2006 67 275 80 10.1016/j.urology.2005.08.017 16461077

[B39] Thüroff JW Bunke B Ebner A Faber P de Geeter P Hannappel J Heidler H Madersbacher H Melchior H Schäfer W Randomized, double-blind, multicenter trial on treatment of frequency, urgency and incontinence related to detrusor hyperactivity: oxybutynin versus propantheline versus placebo J Urol 1991 145 813 7 2005707 10.1016/s0022-5347(17)38459-8

[B40] Uchida T Tempel D Ridge S Grimes I Smith N US PII study results: efficacy and safety of YM905, a bladder-selective treatment for OAB Int Urogynecol J 2002 13 S12

[B41] Van Kerrebroeck P Kreder K Jonas U Zinner N Wein A Tolterodine once-daily: superior efficacy and tolerability in the treatment of the overactive bladder Urology 2001 57 414 21 10.1016/S0090-4295(00)01113-4 11248608

[B42] Wang A Chih S Chen M Comparison of electric stimulation and oxybutynin chloride in management of overactive bladder with special reference to urinary urgency: A randomized placebo-controlled trial Urology 2006 68 999 1004 10.1016/j.urology.2006.05.038 17113893

[B43] Yamaguchi O Marui E Kakizaki H Itoh N Yokota T Okada H Ishizuka O Ozono S Gotoh M Sugiyama T Seki N Yoshida M Japanese Solifenacin Study Group: Randomized, double-blind, placebo-and propiverine-controlled trial of the once-daily antimuscarinic agent solifenacin in Japanese patients with overactive bladder BJU Int 2007 100 579 87 10.1111/j.1464-410X.2007.07031.x 17669143

[B44] Zinner N Gittelman M Harris R Susset J Kanellos A Auerbach S Trospium chloride improves overactive bladder symptoms: a multicenter phase III trial J Urol 2004 171 2311 5 10.1097/01.ju.0000127742.73136.0c 15126811

[B45] Walach H Sadaghiani C Dehm C Bierman D The therapeutic effect of clinical trials: understanding placebo response rates in clinical trials – a secondary analysis BMC Med Res Methodol 2005 5 26 16109176 10.1186/1471-2288-5-26 PMC1201145

[B46] Ramsay IN Thou M A randomised, double-blind, placebo controlled trial of pelvic floor exercises in the treatment of genuine stress incontinence Neurourol Urodyn 1990 9 398 9

[B47] Hunskaar S Arnold EP Burgio K Diokno AC Herzog AR Mallett VT Epidemiology and natural history of urinary incontinence Int Urogynecol J Pelvic Floor Dysfunct 2000 11 5 301 19 10.1007/s001920070021 11052566

[B48] Egger M Smith GD Schneider M Minder C Bias in meta-analysis detected by a simple, graphical test BMJ 1997 315 629 634 9310563 10.1136/bmj.315.7109.629 PMC2127453

